# Mn Inhibits GSH Synthesis via Downregulation of Neuronal EAAC1 and Astrocytic xCT to Cause Oxidative Damage in the Striatum of Mice

**DOI:** 10.1155/2018/4235695

**Published:** 2018-08-30

**Authors:** Xinxin Yang, Haibo Yang, Fengdi Wu, Zhipeng Qi, Jiashuo Li, Bin Xu, Wei Liu, Zhaofa Xu, Yu Deng

**Affiliations:** ^1^Department of Environmental Health, School of Public Health, China Medical University, Shenyang 110122, China; ^2^Department of Occupational Diseases, Linyi People's Hospital, Shandong 276000, China

## Abstract

Excessive manganese (Mn) can accumulate in the striatum of the brain following overexposure. Oxidative stress is a well-recognized mechanism in Mn-induced neurotoxicity. It has been proven that glutathione (GSH) depletion is a key factor in oxidative damage during Mn exposure. However, no study has focused on the dysfunction of GSH synthesis-induced oxidative stress in the brain during Mn exposure. The objective of the present study was to explore the mechanism of Mn disruption of GSH synthesis via EAAC1 and xCT *in vitro* and *in vivo*. Primary neurons and astrocytes were cultured and treated with different doses of Mn to observe the state of cells and levels of GSH and reactive oxygen species (ROS) and measure mRNA and protein expression of EAAC1 and xCT. Mice were randomly divided into seven groups, which received saline, 12.5, 25, and 50 mg/kg MnCl_2_, 500 mg/kg AAH (EAAC1 inhibitor) + 50 mg/kg MnCl_2_, 75 mg/kg SSZ (xCT inhibitor) + 50 mg/kg MnCl_2_, and 100 mg/kg NAC (GSH rescuer) + 50 mg/kg MnCl_2_ once daily for two weeks. Then, levels of EAAC1, xCT, ROS, GSH, malondialdehyde (MDA), protein sulfhydryl, carbonyl, 8-hydroxy-2-deoxyguanosine (8-OHdG), and morphological and ultrastructural features in the striatum of mice were measured. Mn reduced protein levels, mRNA expression, and immunofluorescence intensity of EAAC1 and xCT. Mn also decreased the level of GSH, sulfhydryl, and increased ROS, MDA, 8-OHdG, and carbonyl in a dose-dependent manner. Injury-related pathological and ultrastructure changes in the striatum of mice were significantly present. In conclusion, excessive exposure to Mn disrupts GSH synthesis through inhibition of EAAC1 and xCT to trigger oxidative damage in the striatum.

## 1. Introduction

Manganese is an essential micronutrient in the body; however, chronic overexposure can cause extrapyramidal dysfunction, such as Parkinson's disease (PD), widely referred to as manganism [1]. The level of environmental Mn pollution in China is quite high. According to the *Chinese Bulletin of Environmental Status in 2016*, which is published by the Ministry of Ecology and Environment of People's Republic of China, Mn levels heavily exceeded *drinking water sanitary standard* in groundwater and tap water in China, and the risk of water pollution in China involves 18 provinces or areas and affects nearly 310 million people [2]. Although the equipment necessary to remove Mn and iron from drinking water has been installed on a large scale in China, Mn pollution still exists due to insufficient equipment maintenance. Moreover, gasoline with added methylcyclopentadienyl Mn tricarbonyl (MMT) has been used since 2000, and despite the fact that gasoline with limited Mn was introduced on 1 January 2018, nearly 17 years of accumulation, together with emissions from mining and smelting, mean that Mn pollution in the atmosphere is still widespread.

Recent studies suggest that Mn is able to move across the blood-brain barrier (BBB) and accumulates predominantly in the striatum [3, 4]. The neurodegenerative mechanisms caused by Mn are related to dopamine (DA) system dysfunction, mitochondrial injury, glutamate (Glu) excitotoxicity, and oxidative stress [5]. In fact, dopamine depletion, mitochondria injury, or Glu excitotoxicity all have a relationship with oxidative stress. Therefore, it can be inferred that oxidative stress plays an important role in Mn neurotoxicity. However, the mechanisms of Mn-induced oxidative stress are still under investigation. GSH, as an important nonprotein sulfhydryl compound, plays many important roles in living cells, such as antioxidation, detoxification, scavenging of reactive oxygen species (ROS), and neutralization of organic hydroperoxides [6, 7]. GSH also plays a pivotal role in modulating Mn toxicity [8–10]. The levels of GSH in brain decline with aging [11], and lipids with unsaturated fatty acids that make up the brain are often the target of lipid peroxidation and are vulnerable to oxidative stress [12]. To date, there are numerous studies focusing on GSH depletion during manganism; however, these studies rarely concern the dysfunction of GSH synthesis caused by Mn exposure.

It is known that GSH is a tripeptide composed of Glu, cysteine, and glycine. Cysteine is the rate-limiting substrate in GSH synthesis, especially in neurons [13]. Excitatory amino acid carrier 1 (EAAC1) is able to import Glu and cysteine into the cell, is mainly expressed in neurons, and plays an important role in neuronal GSH synthesis [14]. Mature neurons utilize extracellular cysteine, not cystine, for GSH synthesis, as mature neurons show little or no cystine transporters [15]. Cystine is formed by the oxidation of two cysteines with a disulfide bond, and cysteine is oxidized into cystine in the extracellular milieu [14]. In astrocytes, system Xc^−^ functions as an antiporter that uptakes cystine into cells in exchange for intracellular Glu in a 1 : 1 ratio [16]. This transporter consists of two subunits, xCT and 4F2hc; the former transporter is responsible for transporting activity and substrate specificity, and 4F2hc, the heavy chain, is thought to target the transporter to plasma membrane [17]. xCT is widely located in the brain, where it is expressed by astrocytes [18, 19]. It is recognized that GSH synthesis in neurons is dependent on the expression of xCT in astrocytes [20], as astrocytes contain higher levels of GSH than neurons, and astrocytes release significant amounts of GSH into the extracellular medium [21]. Extracellular GSH, and its metabolites, can generate cysteine, which is then taken up by neurons through EAAC1 for GSH synthesis [22]. The synthesis of GSH in neurons and astrocytes is dependent on the rate of Glu/cysteine exchange and is currently being targeted for numerous disorders of the central nervous system (CNS), which indicates that manganism may have some relationship with the function of EAAC1 and xCT.

The present study was undertaken to test whether excessive treatment with MnCl_2_ can cause EAAC1 and xCT dysfunction that eventually induces GSH depletion and oxidative stress. Therefore, the present study first observed the effects of MnCl_2_ exposure on primary neuronal EAAC1 and astrocytic xCT *in vitro* and investigated their roles in GSH synthesis and oxidative damage by using the EAAC1 inhibitor L-aspartate-beta-hydroxamate (AAH), the xCT inhibitor sulfasalazine (SSZ), and the GSH rescuer N-acetylcysteine (NAC). Then, a mice model was used to verify the effects of EAAC1 and xCT on GSH synthesis and oxidative damage in the striatum *in vivo*.

## 2. Materials and Methods

### 2.1. Reagent and Chemicals

Manganese chloride (MnCl_2_·4H_2_O), L-aspartate-beta-hydroxamate (AAH), sulfasalazine (SSZ), N-acetylcysteine (NAC), 5′5-dithiobis-(2-nitrobenzoic acid) (DTNB), tetraethoxypropane, and folin phenol reagent were obtained from Sigma-Aldrich Chemicals (St Louis, MO, USA). Cell Counting Kit-8, Annexin V/PI apoptosis detection kit, Cy3-labeled donkey anti-goat IgG (H+L), and DAPI staining solution were provided by the Beyotime Institute of Biotechnology (Shanghai, China). Analysis kits of sulfhydryl and carbonyl were provided by the Jiancheng Bioengineering Institute (Nanjing, China). Trizol reagent was provided by the TaKaRa Biotechnology Co., Ltd (Dalian, China). Real-time PCR kit and primer design tool were provided by the Promega Biotech Co., Ltd (Beijing, China). Goat polyclonal antibodies against EAAC1 and xCT, mouse monoclonal antibody against *β*-actin, and rabbit monoclonal antibody against 8-OHdG were provided by the Santa Cruz Biotechnology, Inc. (Santa Cruz, CA, USA). Horseradish peroxidase (HRP) conjugated anti-goat secondary antibody, HRP conjugated anti-mouse secondary antibody, and biotinylated goat anti-rabbit IgG were provided by the Santa Cruz Biotechnology, Inc. (Santa Cruz, CA, USA). Streptavidin-biotin complex (SABC) immunohistochemistry kit and diaminobenzidine (DAB) were provided by the Boster Biochemical Reagent Co., Ltd. (Wuhan, China). Additional chemicals were purchased from local chemical suppliers. All chemicals were analytical grade and the highest pharmaceutical grade.

### 2.2. Primary Neurons and Astrocyte Culture

The primary neuronal culture was prepared using a method described previously [23, 24]. Striata were isolated from neonatal (0–24 h) mice brains and cut into 2 mm pieces under the light microscope. The tissue chunks were then suspended in 10 mL of 0.125% (w/v) trypsin solution (pH 7.4) and placed in a shaking water bath for 20 min at 37°C. The dissociated cells were diluted with DMEM containing 20% (v/v) FBS and centrifuged at 125*g* for 5 min. The cell pellets were resuspended in DMEM containing 10% (v/v) FBS. The whole solution was filtered through stainless steel (200 mesh, hole-width 95 *μ*m). The viability of the dissociated cells was determined by Trypan blue exclusion (>95%). The cell suspension was plated at 5–10^5^ cells/mL on six-well dishes precoated with poly-L-lysine and incubated at 37°C in a 95% humidified atmosphere of 5% CO_2_ up to 24 h. For neurons, the culture medium was changed to Neurobasal™-A Medium (1x) Liquid without Phenol Red and supplemented with B27, 100 U/mL penicillin/streptomycin and L-glutamine (0.5 mM). Half of the culture medium was changed every 3 days. These cells were then treated with cytosine arabinoside (5 *μ*M) on the third day for 48 h to inhibit the division of nonneuronal cells. After 7 days of culture, primary cultures yielded more than 95% neurons, as determined by neuron-specific enolase (NSE) immunostaining. For astrocytes, when the cells had almost reached confluence, 0.25 mM dibutyryl cyclic AMP (dBcAMP) was added to the medium. Cells that showed more than 95% positive astrocyte marker glial fibrillary acidic protein (GFAP) staining were used for this experiment.

### 2.3. Animals

All animal studies were approved by the Scientific Research Committee of China Medical University and have been conducted in accordance with the Chinese National Guidelines for the Care and Use of Laboratory Animal in animal experiments. All efforts were made to minimize the number of animals used and their suffering.

The study was performed both on newborn (one day old for primary cell extraction) and adult (30 ± 2 g weight at the beginning of experiment; *N* = 112; equal numbers of male and female) Kunming mice from the Laboratory Animal Center of China Medical University, Shenyang, China (SPF grade, Certificate No. SCXK2013-0001). They were housed in plastic cages in a climate-controlled animal room (temperature, 24°C ± 1°C; humidity, 55% ± 5%) with 12 hr light/dark cycle and allowed free access to food (Certified diet, Laboratory Animal Center of China Medical University, Shenyang, China) and water.

### 2.4. Experimental Design and Treatments

*In vitro*: groups exposed to different concentrations of Mn, neurons, and astrocytes were seeded in plates or dishes exposed to 0, 100, 200, and 400 *μ*M MnCl_2_ for 24 h. In the AAH treatment group, neurons were treated with 250 *μ*M AAH and 400 *μ*M MnCl_2_ for 24 h. In the SSZ treatment group, astrocytes were treated with 200 *μ*M SSZ + 400 *μ*M MnCl_2_ for 24 h, and in the NAC-treated group, neurons and astrocytes were treated with 1 mM NAC + 400 *μ*M MnCl_2_ for 24 h.

*In vivo*: all the mice were divided into seven groups by weight randomly, *n* = 16 for each group. The first to seventh groups were control, low, intermediate, and high dose of MnCl_2_, AAH+MnCl_2_, SSZ+MnCl_2_, and NAC+MnCl_2_ group, respectively. Mice in the first to fourth group were administrated with 0.9% NaCl subcutaneously (s.c.). Mice in the fifth or seventh group were s.c. administrated with 500 mg/kg AAH [25], 75 mg/kg SSZ [26–28], and 100 mg/kg NAC [29], respectively. After 1 h, animals in the first group were intraperitoneally (i.p.) injected with 0.9% NaCl; mice in the second to seventh group were i.p. injected with 12.5, 25, 50, 50, 50, and 50 mg/kg MnCl_2_, respectively. The volume of administration was 5 mL/kg. The injection was given every day, for 2 weeks. Mice in each group were sacrificed by decapitation after anesthesia 24 h after the last injection. The brain capsule of six mice in each group was removed on ice bath, and the striatum separated and prepared as a 10% homogenate in order to detect the levels of GSH, MDA, and ROS. Total RNA and protein was extracted from the other four striata for testing objective mRNA and protein levels. The striata from four mice in each group were cut into 5 mm thick tissue pieces and fixed in 4% paraformaldehyde in order to observe pathological changes by HE staining and the level of EAAC1, xCT, and 8-OHdG by immunohistochemical analysis. The striata of the remaining two mice in every group were fixed in 2.5% glutaraldehyde buffer to observe the ultrastructural features. All the substance levels and enzyme activities were normalized to the protein content, which was measured following the method of Lowry et al. [30], using bovine serum albumin (BSA) as standard.

### 2.5. Western Blotting Analysis of EAAC1 and xCT

Total protein was extracted from the striatum using RIPA buffer (10 mM Na_2_HPO_4_, pH 7.2, 150 mM NaCl, 1% sodium deoxycholate, 1% Nonidet P-40, 0.1% SDS) containing protease inhibitors (1 mM phenylmethylsulfonyl fluoride, 0.2 mM 1,10-phenanthroline, 10 *μ*g/mL pepstatin A, 10 *μ*g/mL leupeptin, 10 *μ*g/mL aprotinin, and 10 mM benzamidine). Protein concentrations were determined by BCA reagent. Equal amounts of protein (40 *μ*g) were separated by 8% polyacrylamide gel electrophoresis and transferred to polyvinylidene difluoride (PVDF) membranes (Millipore, Ternicula, CA). PVDF membranes were blocked at room temperature for 1 h in Tris-buffered saline with 0.1% Tween 20 (TBST) containing 5% bovine serum albumin fraction V and then incubated with the primary antibody overnight at 4°C. The primary antibodies were performed as follows: EAAC1 (1 : 500), xCT (1 : 500), and *β*-actin (1 : 1000). After washing with TBST, the membranes were incubated with the secondary anti-rabbit or anti-mouse HRP-conjugated antibodies (1 : 5000) for 1 h at room temperature. Bands were visualized using the ECL kit. To quantify the staining, densitometric analysis was performed using the Flurchen V2.0 Stand Alone software. The relative intensity of each protein sample was normalized to *β*-actin.

### 2.6. Quantitative Real-Time PCR Analysis

mRNA expression levels were analyzed using a real-time reverse-transcription polymerase chain reaction assay. Total RNA was extracted using the Trizol method (TransGen, Beijing, China) and redissolved in RNase-free water (TaKaRa). The absorbance of the RNA solution was determined using a NanoPhotometer (Implen, Eppendorf, Germany) at 260 and 280 nm. A260/A280 ratios were between 1.7 and 2.1. First strand cDNA was synthesized from 1 *μ*g of total RNA by reverse transcriptase using a PrimeScript™RT reagent Kit (TaKaRa) and oligo (dT) primers (TaKaRa) according to the manufacturer's protocol. Real-time quantitative PCR (RT-PCRq) was performed using SYBR® Premix Ex Taq™ II kit (TaKaRa) using an ABI 7500 Real-Time PCR System (Applied Biosystems, Waltham, MA). Two microliters of template cDNA were added to a final reaction mixture volume of 20 *μ*L. Real-time PCR cycle parameters were 30 s at 95°C followed by 40 cycles of denaturation at 95 for 5 s, annealing at 60°C for 34 s, and elongation at 72°C for 20 s. The sequences of the specific sets of primers for EAAC1, xCT, and *β*-actin used in this study are given in Table 1. The expression of selected genes was normalized to the *β*-actin gene, which was used as an internal housekeeping control. For relative quantification of the tested target genes, we used the comparative CT method (ΔΔCT). All the real-time PCR experiments were performed in quadruplicate, and data were expressed as the mean of at least three independent experiments.

### 2.7. Immunofluorescence Analysis of EAAC1 and xCT

Immunofluorescence was used to examine the localization and number of EAAC1- and xCT-positive cells in neurons and astrocytes, respectively. The collected cells were grown in six-well plates that were covered with a cover glass and cultured in an incubator (37°C in a 95% humidified atmosphere of 5% CO_2_). Cells were removed after cell adherence, washed with PBS for 5 min, placed in stationary liquid (absolute ethyl alcohol : chloroform : glacial acetic acid = 6 : 3 : 1) for 20 min of fixation, followed by natural drying, and washing with PBS buffer (0.01 M, pH 7.2) for 5 min 3 times. Cells were permeabilized and blocked in 10% FBS containing 3% (w/v) bovine serum albumin and 0.1% Triton X-100 for 1 h at room temperature in order to avoid unspecific staining, then the sections were incubated with goat polyclonal primary antibodies EAAC1 and xCT (1 : 50) overnight at 4°C. Sections were then incubated with the secondary antibodies (Cy3-labeled donkey anti-goat IgG and Alexa Fluor 488-labeled goat anti-rabbit IgG (1 : 250)) for 2 h at room temperature. After washing with PBS 3 times, the specimens were counterstained by 2-(4-Amidinophenyl)-6-indolecarbamidine dihydrochloride (DAPI) for 5 min. After washing again, the sections were covered with microscopic glass with antifade polyvinylpyrrolidone mounting medium for further study. Images were captured using a BX61 fluorescence microscope (Olympus, Japan).

### 2.8. GSH Assay

GSH levels were determined by reaction with the 5′5-dithiobis-(2-nitrobenzoic acid) (DTNB) colorimetric method [31]. In brief, 0.9 mL of the 10% striatum homogenate was added to 0.1 mL 50% trichloroacetic acid, and samples were centrifuged at 4000 rpm for 10 min. Next, 0.1 mL of supernatant was added to 4.4 mL of 0.1 M PBS and 0.04% DTNB up to total volume of 5 mL (pH 7.4). The absorbance of the solution was measured spectrophotometrically at 412 nm. The concentration of GSH was expressed as *μ*mol/g protein.

### 2.9. Determination of ROS Generation

The striatum was rendered as single cell suspension according to the method of Villalba et al. [32]. Intracellular ROS generation was measured using a flow cytometer with an oxidation-sensitive 2,7-dichlorofluorescin-diacetate (DCFH-DA) fluorescent probe [33].

### 2.10. Apoptosis Analysis

Flow cytometry and an Annexin V/PI apoptosis detection kit were used to assess membrane and nuclear events that occurred during apoptosis. Quadrants were positioned on Annexin V/PI dot plots, allowing live (Annexin V−/PI−), early/primary apoptotic (Annexin V+/PI−), late/secondary apoptotic (Annexin V+/PI+), and necrotic cells (Annexin V−/PI+) to be distinguished. Cells were separated and washed three times with PBS (4°C, pH 7.4) and centrifuged at 2000 rpm for 5 min. 1–5 × 10^5^ cells per sample were used. The supernatant was discarded, and the supplied 0.5 mL binding buffer was used to resuspend the cells. 5 *μ*L PI was added to the cell suspension, and the mixture was incubated for 15 min protected from light at room temperature. Apoptosis was analyzed by flow cytometry (Becton Dickinson, USA).

### 2.11. MDA Assay

The 10% striatum homogenate was added into a reaction mixture containing 0.1 M PBS and 0.1 M FeCl_3_ in a total volume of 1 mL (pH = 7.4). The reaction was stopped by adding 1 mL 10% TCA, followed by 1 mL 0.67% TBA, and all the tubes were placed in 90–100°C water bath for 20 min. Finally, the tubes were centrifuged at 4000*g* for 10 min. The amount of MDA formed in each of the samples was assessed by measuring the optical density of the supernatant at 535 nm using tetraethoxypropane (TEP) as standard. MDA content was expressed as nmol·mg^−1^ protein.

### 2.12. Measurement of Sulfhydryl and Carbonyl

The sulfhydryl and carbonyl contents of the striatum were measured using assay kits, according to the manufacturer's instructions. The sulfhydryl content was expressed as *μ*mol/g protein, and carbonyl as nmol·mg^−1^ protein.

### 2.13. Immunohistochemical Analysis of 8-OHdG

The levels of 8-OHdG were measured using immunohistochemistry. After the striatum was fixed by 4% paraformaldehyde for 24 h, it was dehydrated by a gradient of alcohol and xylene. Then, the tissue was embedded by low-melting-point paraffin and cut using a Leitz 1512 microtome (Leitz Co., Germany) at 5 *μ*m thickness each. For the immunohistochemistry reaction, the slice was rinsed with PBS 3 times, 5 min each, then incubated in 3% (v/v) H_2_O_2_ at room temperature for 10 min, followed by antigen repair and blocking with 10% (v/v) nonimmune goat serum. Twenty minutes later, 100 *μ*L 8-OHdG antibody was added at 1 : 100 dilution 37°C for 30 min after the serum was removed. Then, biotinylated anti-rabbit IgG with 8-OHdG antibody was added after the slices were rinsed for 10 min at 37°C. SABC reagent was added for 10 min at 37°C. Then, the slice was performed with 3,3-diaminobenzidine (DAB) and counterstained with hematoxylin after differentiation with hydrochloric alcohol and finally sealed with neutral gum. The photos were captured by autoexposure microscopy (Olympus, Japan), and the images were analyzed using the Image J graphic analysis system. For two mice in each group, three occasions for each mouse, the integral optical density (IOD) of the 8-OHdG antibody-immune response-positive-cells was calculated.

### 2.14. HE Staining

After the striata were removed and fixed in 4% paraformaldehyde, they were serially dehydrated in graded ethanol and xylene. Tissues were paraffin-embedded and sectioned at 5 *μ*m thickness. Regular hematoxylin and eosin staining was performed for morphological observation with a microscope (Olympus AX-70).

### 2.15. Electron Microscopy Studies

The striatum tissue was fixed in 2.5% glutaraldehyde buffer in 0.1 M PBS. After fixation for 1 h, 1 mm thick slices were made from the tissue. Fixation was prolonged overnight in fresh fixative. For electron microscopy, postfixation of slices in 1% OsO_4_ containing 1.25% potassium ferrocyanide was carried out. Afterwards, tissues were dehydrated in a graded series of acetone and embedded in Spurr resin. Finally, blocks were stained with uranyl acetate and lead citrate and photographed by a JEM-1200EX transmission electron microscope equipped with an UltraScan digital camera.

### 2.16. Statistical Analysis

Measurement of the parameters was conducted in triplicate, and the means from three experiments were used for statistical analysis. Results were presented as mean ± standard deviation. All statistical analysis was performed using the SPSS software, version 18.0. Differences between the means were determined by one-way ANOVA followed by a Student-Newman-Keuls test for multiple comparisons. The differences at either *p* < 0.05 or *p* < 0.01 were considered statistically significant.

## 3. Results

### 3.1. EAAC1 and xCT Expression *In Vitro*

**Figure 1:**
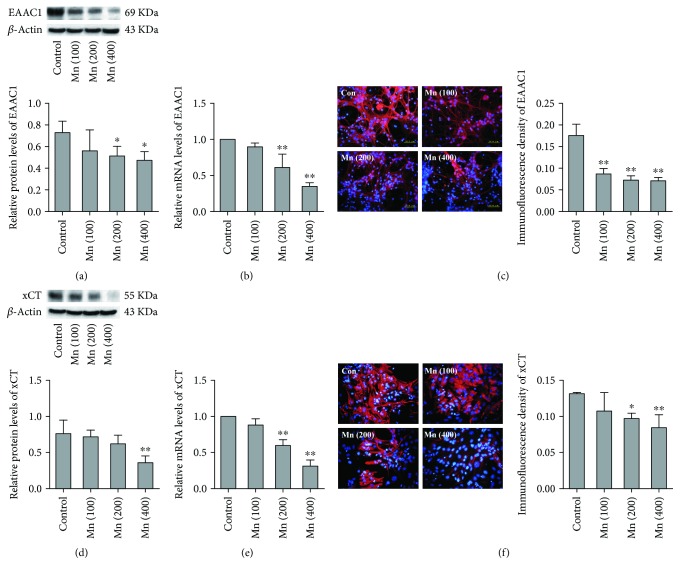
The alteration of EAAC1 protein (a), mRNA (b), and immunofluorescence density (c) in neurons and the changes in xCT protein (d), mRNA (e), and immunofluorescence density (f) in astrocytes after MnCl_2_ exposure are shown in this figure (*in vitro*). Data are expressed as mean ± SD (*n* = 4). Each RNA or protein preparation was run three times by real-time PCR or Western blotting; ^∗^*p* < 0.05, ^∗∗^*p* < 0.01 denote statistical significance compared with control group.

EAAC1 is a transporter that is expressed on neurons, which mediates the transport of Glu and cysteine and plays an important role in GSH synthesis. To explore the alternation of EAAC1 on neurons induced by Mn *in vitro*, we measured the protein levels, mRNA expression, and immunofluorescent intensity of EAAC1. As shown in Figure 1(a), the protein levels of EAAC1 decreased by 29.71% and 35.35% in the 200 and 400 *μ*M MnCl_2_ groups when compared with the control group (*p* < 0.05; *p* < 0.05). Meanwhile, mRNA expression of EAAC1 at 200 and 400 *μ*M MnCl_2_ exposure decreased 39.10% and 65.00% compared with the control (*p* < 0.01; *p* < 0.01).  Figure 1(c) shows immunofluorescent microscopy and the analysis of immunofluorescent intensity of EAAC1 for neurons. It was found that the amount of anti-EAAC1-positive cells (red) diminished sharply after MnCl_2_ exposure. After assay and calculation, the immunofluorescent intensity of EAAC1 in the 100, 200, and 400 *μ*M MnCl_2_ groups decreased significantly compared to the untreated group (*p* < 0.01; *p* < 0.01; *p* < 0.01), especially in the 400 *μ*M MnCl_2_ group where there was an overwhelming reduction of EAAC1 expression.

xCT, a key subunit that is mainly expressed in astrocytes, exchanges intracellular Glu for extracellular cystine. Due to xCT's critical function for GSH synthesis, to confirm whether it can be altered by Mn is of great concern. It was found that Mn inhibited protein levels of xCT on astrocytes (Figure 1(d)). Especially in the 400 *μ*M MnCl_2_ group, the xCT protein level decreased to 52.06% of the control (*p* < 0.01).  Figure 1(e) shows that the mRNA expression of xCT in the 200 and 400 *μ*M MnCl_2_ groups decreased to 39.82% and 68.73% of control, respectively.  Figure 1(f) suggested the amount of anti-xCT-positive cells (red) for astrocytes diminished in a concentration-dependent manner. For the 200 and 400 *μ*M MnCl_2_ groups, the immunofluorescent signal intensity of xCT reduced to 26.47% and 35.31% of control group, respectively (*p* < 0.05; *p* < 0.01).

### 3.2. EAAC1 and xCT Expression *In Vivo*

**Figure 2:**
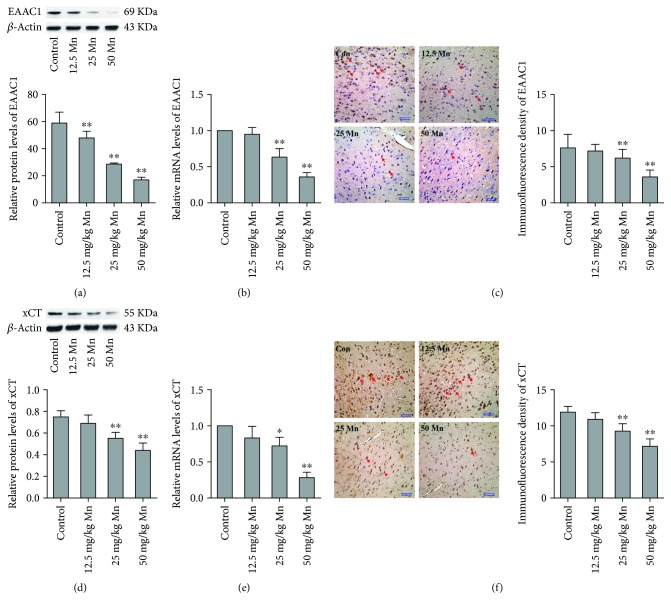
The alteration of EAAC1 protein (a), mRNA (b), and immunofluorescence density (c), xCT protein (d), mRNA (e), and immunofluorescence density (f) in the striatum after MnCl_2_ exposure are shown in this figure (*in vivo*). Data expressed as mean ± SD for four mice in each group. Each RNA or protein preparation was run three times by real-time PCR or Western blotting; ^∗^*p* < 0.05, ^∗∗^*p* < 0.01 denotes statistical significance compared with control group.

Because the complex mechanisms of oxidative stress cannot be fully demonstrated *in vitro*, we performed the same experiments as mentioned above to identify the effects of EAAC1 and xCT in mice striatum. As shown in Figure 2(a), 12.5, 25, and 50 mg/kg MnCl_2_ caused significant decreases in EAAC1 protein levels of 18.63%, 51.31%, and 71.16%, respectively, versus the control in striatum (*p* < 0.01; *p* < 0.01; *p* < 0.01).  Figure 2(b) shows that in the 25 and 50 mg/kg MnCl_2_ groups, EAAC1 mRNA expression decreased approximately 36.75% and 63.99% relative to control counterparts (*p* < 0.01; *p* < 0.01). In Figure 2(c), the EAAC1-positive immunohistochemical staining in the 25 and 50 mg/kg MnCl_2_ groups was less than 37.99% and 48.62% of the control group (*p* < 0.01; *p* < 0.01).

As shown in Figure 2(d), after treatment with 25 and 50 mg/kg MnCl_2_, xCT protein levels were reduced to 26.29% and 36.54% compared with control, respectively, in the striatum (*p* < 0.01; *p* < 0.01).  Figure 2(e) shows that when compared with the control, xCT mRNA expressions decreased of 27.72% and 66.58% in 25 and 50 mg/kg MnCl_2_ groups (*p* < 0.05; *p* < 0.01). xCT positive staining was decreased in a concentration-dependent manner (Figure 2(f)). In the 25 and 50 mg/kg MnCl_2_ groups, the immunohistochemical optical density (IOD) of xCT decreased by 21.90% and 38.89% compared with control (*p* < 0.01; *p* < 0.01).

### 3.3. ROS and GSH Alternation *In Vitro*

As excessive ROS is the source of oxidative stress in the body, we tested whether ROS concentration is altered after treatment with Mn. As demonstrated in Table 2, the exposure of Mn to neurons causes an increase in ROS in a concentration-dependent manner. Compared to the control group, the administration of 200 and 400 *μ*M MnCl_2_ significantly increased ROS concentration by approximately 1.42- and 1.80-fold (*p* < 0.01; *p* < 0.01). Compared to the 400 *μ*M MnCl_2_ group, ROS in the 250 *μ*M AAH pretreated group improved 1.20-fold (*p* < 0.01), while in the 1 mM NAC + 400 *μ*M MnCl_2_ group, ROS was reduced by 36.53% (*p* < 0.01). GSH is the major nonenzymatic antioxidant that scavenges ROS and inhibits oxidative injury. In this study, we observed that GSH levels in neurons were decreased by 22.87%, 34.01%, and 42.97% in the 100, 200, and 400 *μ*M MnCl_2_ groups, respectively, when compared with the untreated group (*p* < 0.05; *p* < 0.01; *p* < 0.01). Cotreatment with 250 *μ*M AAH causes GSH to decrease to 35.86% in the 400 *μ*M MnCl_2_ group (*p* < 0.05); however, cotreatment with NAC elevated it 1.66-fold (*p* < 0.05).  Table 3 reveals ROS and GSH changes in astrocytes after treatment with MnCl_2_ or cotreatment with SSZ or NAC. ROS levels in the 200 and 400 *μ*M MnCl_2_ groups were 1.33 and 1.55 times higher than SAL-SAL group (*p* < 0.01; *p* < 0.01). Importantly, compared with the SAL-Mn group, ROS increased 1.10 times in the SSZ + Mn group (*p* < 0.01) but decreased 26.30% in the NAC + Mn group (*p* < 0.01). In contrast, GSH was significantly decreased in the 200 and 400 *μ*M MnCl_2_ groups compared to the control group (*p* < 0.05; *p* < 0.01). Meanwhile, GSH decreased to 37.21% in the SSZ-Mn group while it increased to 1.27-fold in the NAC-Mn group compared with the 400 *μ*M MnCl_2_ group (*p* < 0.05, *p* < 0.05).

### 3.4. ROS and GSH Alternation *In Vivo*

Due to the complex oxidative mechanisms in the body, we also focused on the alternation of GSH and ROS levels after treatment with Mn in the striatum of mice *in vivo*. As Table 4 shows, administration of 12.5, 25, and 50 mg/kg MnCl_2_ elevated intracellular ROS levels to 1.85, 1.96, and 2.15 times in the striatum when compared with the saline group (*p* < 0.01; *p* < 0.01; *p* < 0.01). In the 500 mg/kg AAH and 75 mg/kg SSZ groups, the ROS levels were elevated by 1.52- and 1.61-fold compared to the 50 mg/kg MnCl_2_ group (*p* < 0.05; *p* < 0.05), while in the 100 mg/kg NAC + 50 mg/kg MnCl_2_ group, ROS level reduced to 11.65% (*p* < 0.01). Conversely, GSH levels in the MnCl_2_-treated group (12.5, 25, and 50 mg/kg) reduced 17.18%, 38.98%, and 55.58% comparing with the saline-treated group (*p* < 0.01; *p* < 0.01; *p* < 0.01). In addition, coadministration with AAH and SSZ had a higher GSH reduction level than the MnCl_2_-treated group (*p* < 0.05; *p* < 0.05); however, coadministration with NAC increased GSH 1.43-fold compared with the MnCl_2_-treated group (*p* < 0.05).

### 3.5. Cell Activity and Apoptosis in Neurons and Astrocytes

**Figure 3:**
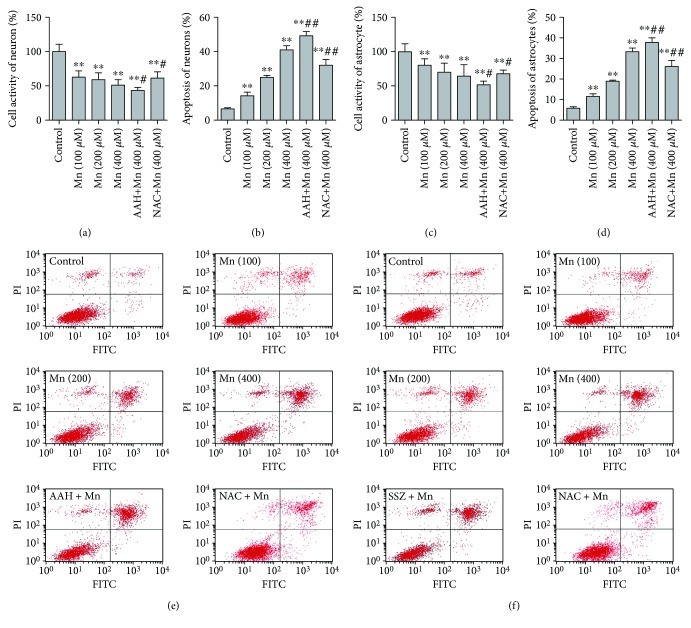
Alterations of cell activity and apoptosis after MnCl_2_ exposure or AAH, SSZ, and NAC coadministration. (a, b) Cell activity and apoptosis rate of neurons. (c, d) Cell activity and apoptosis rate of astrocytes. (e) Apoptosis results of neurons. (f) Apoptosis results of astrocytes. Data expressed as mean ± SD. *n* = 10 for cell activity; *n* = 4 for cell apoptosis rate in each group. ^∗∗^*p* < 0.01 denote statistical significance compared with the control group; ^#^*p* < 0.05, ^##^*p* < 0.01 denote statistical significance compared with the 400 *μ*M MnCl_2_ group.

Cell activity and apoptosis rate of neurons and astrocytes were detected after exposure to a series of concentrations of MnCl_2_ or cotreatment with AAH, SSZ, and NAC. Whether neurons or astrocytes, cell activity rate gradually decreased in a Mn concentration-dependent manner; meanwhile, cotreatment with AAH to neurons or SSZ to astrocytes both induced cell activity decreases significantly compared with treatment with SAL-Mn (*p* < 0.05; *p* < 0.05). Inversely, coadministration with NAC protected the increased cell activity induced by Mn in neurons and astrocytes (*p* < 0.05; *p* < 0.05) (Figures 3(a)–3(b)). The apoptosis rate of primary cultured neurons and astrocytes was measured. Compared with the control group, elevated cell apoptosis rates of neurons (Figure 3(c)) and astrocytes (Figure 3(d)) were observed with the elevation of Mn. In addition, cotreatment with AAH to neurons or SSZ to astrocytes all elevated the cell apoptosis rate significantly compared with the treatment with 400 *μ*M Mn (*p* < 0.01; *p* < 0.01). Conversely, coadministration with NAC can prevent the increased cell apoptosis rate induced by Mn in neurons and astrocytes (*p* < 0.01; *p* < 0.01).

### 3.6. Oxidative Injury in the Striatum

**Figure 4:**
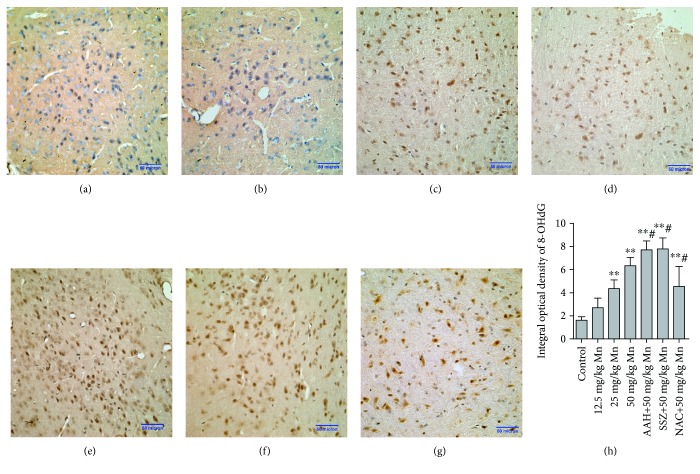
Immunohistochemistry assay of 8-OHdG in the striatum after MnCl_2_ exposure or AAH, SSZ, and NAC cotreatment. Photographs of control (a), 12.5 mg/kg MnCl_2_ (b), 25 mg/kg MnCl_2_ (c), 50 mg/kg MnCl_2_ (d), 500 mg/kg AAH + 50 mg/kg MnCl_2_ (e), 75 mg/kg SSZ + 50 mg/kg MnCl_2_ (f), and 100 mg/kg NAC + 50 mg/kg MnCl_2_ (g). The sections were stained with SABC, and the magnification was set at ×40. Data are shown as mean ± SD for four mice per group. The effect of MnCl_2_ or its coadministration with AAH, SSZ, and NAC on the 8-OHdG integral optical density (h) is also shown. ^∗∗^*p* < 0.01 denotes statistical significance compared with the control group; ^#^*p* < 0.05 denotes statistical significance compared with the 50 mg/kg MnCl_2_ group.

We measured all changes in MDA and carbonyl as well as sulfhydryl after treatment with different concentrations (0, 12.5, 25, and 50 mg/kg) of MnCl_2_ or cotreatment with AAH, SSZ, or NAC to determine Mn-triggered oxidative injury of lipid and protein (Table 5). It was found that MnCl_2_ increased levels of MDA and carbonyl in a dose-dependent manner, of which MDA and carbonyl in the 50 mg/kg MnCl_2_ group were elevated to 2.74- and 2.66-fold of the control (*p* < 0.01; *p* < 0.01). While sulfhydryl levels were decreased significantly after treatment with MnCl_2_, they were reduced to 22.51% and 39.06% in the 25 and 50 mg/kg MnCl_2_ groups, respectively, compared with the untreated group (*p* < 0.01; *p* < 0.01). It was also found that pretreatment of AAH and SSZ increased MDA by 1.42- and 1.36-fold (*p* < 0.05; *p* < 0.05), increased carbonyl 1.24- and 1.19-fold (*p* < 0.05; *p* < 0.05), but decreased sulfhydryl to 28.15% and 39.18% (*p* < 0.05; *p* < 0.05), respectively, compared with the treatment with 50 mg/kg MnCl_2_. However, pretreatment with NAC decreased MDA and carbonyl 29.51% and 24.42% (*p* < 0.05; *p* < 0.01) and increased sulfhydryl 1.31-fold (*p* < 0.01) when compared with the 50 mg/kg MnCl_2_ group. The formation of 8-OHdG is an indicator of oxidative damage to DNA. Generation of 8-OHdG was observed in the MnCl_2_ group (12.5, 25, and 50 mg/kg) or the AAH, SSZ, and NAC coadministration groups (Figure 4(a)–4(g)). In addition, the 8-OHdG immunohistochemical staining was quantitatively evaluated by measuring the IOD in the Image J graphic analysis system (Figure 4(g)) The IOD of 8-OHdG in the 25 and 50 mg/kg MnCl_2_ groups was increased by 2.70- and 3.91-fold of the control (*p* < 0.01; *p* < 0.01). When compared to the 50 mg/kg MnCl_2_ group, the IOD of 8-OHdG was elevated 1.23- and 1.22-fold in the AAH and SSZ cotreated groups, respectively (*p* < 0.05; *p* < 0.05), but decreased by 28.23% in the NAC cotreated group (*p* < 0.05).

### 3.7. Morphological Observation in the Striatum

**Figure 5:**
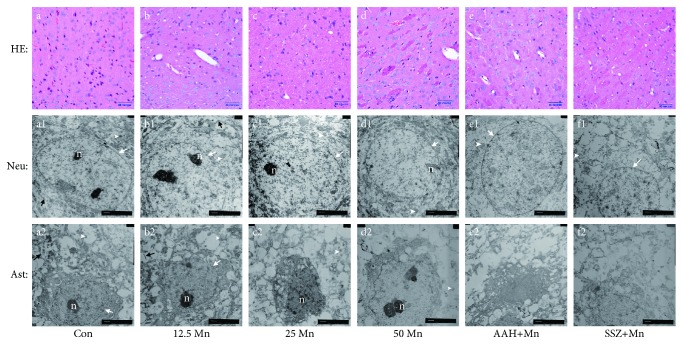
Light and electron microphotographs show the pathological changes of the striatum tissue after exposure to MnCl_2_ or cotreatment with AAH or SSZ. The magnification of the sections stained with HE was ×40. Electron microphotograph magnification was ×4000. White arrows represent the nuclear membrane; n representative nuclei; white arrowheads represent mitochondria; black arrows represent endoplasmic reticulum.

HE staining was used to confirm the effect of MnCl_2_ and AAH or SSZ exposure on the morphological changes of the striatum.  Figure 5(a) presents the normal microstructure in the striatum: neurocyte nuclei stained clearly, and tissue was intact and regular. After treatment with 12.5 mg/kg MnCl_2_, a part of the neurons' nuclei swelled, and tissue arrangement changed slightly (Figure 5(b)). In the 25 mg/kg MnCl_2_ group, degeneration of structure and a decrease number of neurons were observed (Figure 5(c)). After treatment with 50 mg/kg MnCl_2_, serious cell body swelling and interstitial vacuolation appeared (Figure 5(d)). In the pretreated 500 mg/kg AAH or 75 mg/kg SSZ group (Figures 5(e) and 5(f)), the neuropathological injury was worse, and there was greater neuronal loss and a higher number of dead cells accompanying the disordered arrangement and adipose degeneration.

Electron microphotographs showed the ultrastructural changes of neurons and astrocytes in the striatum following Mn exposure and 500 mg/kg AAH or 75 mg/kg SSZ pretreatment.  Figure 5(a1) represents the normal neuronal ultrastructural in the striatum of the control group. The nuclei were approximately circular and contain integrated nuclear membrane, mitochondria, and endoplasmic reticulum and have normal structure and are not swollen. When treated with 12.5 mg/kg MnCl_2_, the nucleolus appeared collapsed, and the membrane integrity was damaged slightly (Figure 5(b1)). After administration with 25 mg/kg MnCl_2_, a serious membrane integrity loss and intense chromatin condensation were present accompanying swollen mitochondria and swollen endoplasmic reticulum (Figure 5(c1)). In the 50 mg/kg MnCl_2_ and 500 mg/kg AAH or 75 mg/kg SSZ pretreatment groups, the neurons exhibited shrinkage of the nucleus accompanying a missing nucleus and swollen and degraded organelles (Figures 5(d1), 5(e1), and 5(f1)).  Figure 5(a2) shows the normal astrocytic ultrastructure in the striatum of the control group, showing an integrated nuclear membrane, clear nucleus, and well-distributed chromatin. In the 12.5 and 25 mg/kg MnCl_2_ groups, the astrocytic ultrastructure exhibited shrinkage of the nucleus, collapse of the nucleus, and disordered cytoplasm arrangement, accompanying swollen mitochondria (Figures 5(b2) and 5(c2)). After exposure to 50 mg/kg MnCl_2_ or pretreatment with 500 mg/kg AAH or 75 mg/kg SSZ, the ultrastructure damage was worse with missing nucleus, intense chromatin condensation, unrecognizable organelle fragments, and cytoplasm vacuolization formation (Figures 5(d2), 5(e2), and 5(f2)).

## 4. Discussion

**Figure 6:**
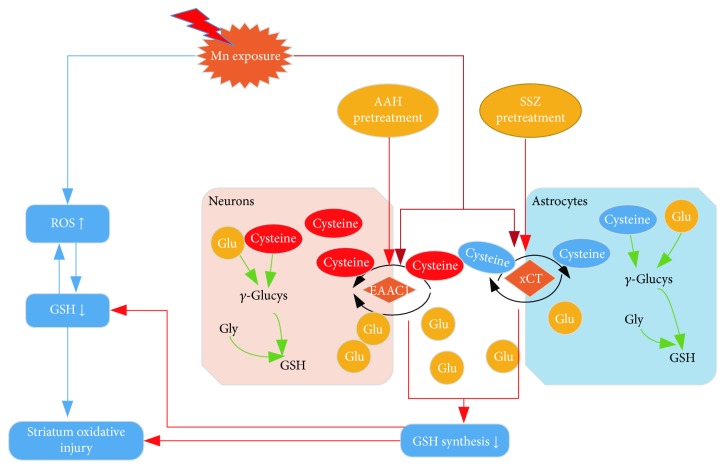
Model showing potential mechanisms of Mn-triggered oxidative damage in the striatum. Cysteine is a limiting substrate for GSH synthesis; EAAC1 and xCT are the transporters located in neurons and astrocytes, respectively. By inhibiting cysteine import, Mn disrupts GSH synthesis.

Oxidative stress plays a crucial role in the pathophysiology of manganism, and the tripeptide GSH is the main antioxidant molecule in the brain. Our study was designed to explore the mechanism of Mn-induced oxidative stress and the role that EAAC1 and xCT play in GSH synthesis (Figure 6). In the brain, *γ*-glutamylcysteine synthase (*γ*-GCS) catalyzes the formation of *γ*-glutamyl-l-cysteine (*γ*-GC) from Glu and cysteine/cystine, and then glutathione synthase catalyzes the formation of GSH from *γ*-GC and Gly; cysteine is a limiting substrate [34, 35]. EAAC1 and xCT play major roles on cysteine/cystine uptake into the neurons and astrocytes [36]. In the current study, it was found that Mn inhibits protein levels of EAAC1 in neurons and xCT of astrocytes in a concentration-dependent manner *in vitro*. In addition, Mn also decreased EAAC1 and xCT mRNA and immunofluorescence density level. (Figure 1). In addition, the expression of EAAC1 and xCT *in vivo* was roughly the same as *in vitro* (Figure 2). Our data suggest that Mn may affect the transcription and protein expression of EAAC1 and xCT.

The formation of ROS is of great importance regarding neuronal damage, structural destruction, and functional disorder of the cells [37, 38]. Our current study found that Mn exposure stimulated the generation of ROS. Cotreatment with AAH, a blocker of EAAC1, increased ROS greatly in cultured neurons and mice striatum. Moreover, coadministration of SSZ, a xCT selective inhibitor, also elevated ROS significantly in cultured astrocytes and mice striatum. Our data indicated that inhibition of EAAC1 or xCT can aggravate Mn-induced ROS increase. The mechanism of Mn-induced neurodegenerative disorder is considered to be relevant to causing oxidative stress by either overproduction of ROS and/or inhibition of antioxidants [39]. Aoyama et al. found that both CA1 and CA3 neurons display a significant increase of ROS and loss of GSH in EAAC1 knockout mice [40]. Dai et al. found that RNAi direct silencing of xCT in primary effusion lymphoma cells can increase intracellular ROS production [41].

GSH is the major nonenzymatic antioxidant that scavenges ROS and inhibits oxidative stress in the CNS [35]. Because GSH poorly penetrates the BBB, it mainly depends on synthesis in the brain itself [22]. In our study, the level of GSH was decreased after exposure to Mn and may be caused by increased oxidative stress and/or decreased GSH synthesis. Previous studies have mainly focused on GSH depletion; however, inhibition of GSH synthesis also contributed greatly to oxidative injury. As expected, blocking EAAC1 or xCT does decrease GSH levels and increase ROS formation compared to high-dose Mn. Preincubation with AAH, a relatively selective EAAC1 inhibitor, in midbrain slice cultures blunted cysteine increase significantly, suggesting that EAAC1 is the primary cysteine transporter [42]. EAAC1 is mainly expressed in mature neurons and involved in cysteine uptake [14]. Mature neurons utilize extracellular cysteine, not cystine, for GSH synthesis, as mature neurons do not have xCT cystine transporters [15]. Therefore, EAAC1 is crucial for GSH synthesis and ROS scavenging. Whereas astrocytes mainly utilize cystine through transporter xCT [15], SSZ mainly blocks the functions of xCT but does not affect its expression or cellular location [41]. Astrocytes play a more important antioxidant role than neurons, not only as GSH levels are lower in neurons than in astrocytes [43] but also because the neurons' maintenance of stable concentrations of GSH depends on astrocytes [44, 45]. So if xCT is inhibited, the effects of oxidative damage may be more severe; however, the real mechanism is more complex. It was found that an extracellular excess of Glu leads to GSH deficiency and oxidative cell death through inhibition of cystine uptake in pure astrocyte cultures [46], whereas astrocytes cultured with neurons were poorly affected by Glu as neuronal factors may induce EAATs expression in astrocytes as a replacement for system Xc^−^ [47–49]. In fact, high concentrations of Glu, which can act as a neurotoxicant, also contributed to ROS increase [50]. Our previous experiments also showed Glu levels in the striatum were increased after treatment with Mn [37]. Trotti et al. found oxidative stress is likely involved in Mn-induced dysregulation of astrocytic Glu transporters since oxidative stress inhibits Glu transporter function [51]. EAAC1 and xCT also act as Glu transporters, and their inhibition may cause Glu excitatory toxicity and aggravate oxidant stress.

Since Mn increased ROS levels and decreased GSH, neuron and astrocyte cell activity and apoptosis were also measured. It was found that Mn decreased cell activity while increasing cell apoptosis in a concentration-dependent manner. In the AAH + Mn or SSZ + Mn group, cell activity was lower while apoptosis rate was higher than in the high-dose Mn group. The results suggested administration of Mn caused neuropathological changes, such as neuron loss, necrosis, and apoptosis, and blocked the expression of EAAC1 and xCT in the striatum, greatly impacting cell survival and antioxidant function. NAC is the acetylated precursor of L-cysteine, which itself is a precursor of GSH [52, 53]; it could rescue both neurons and astrocytes against oxidative stress by increasing intracellular GSH levels independent of EAAC1 or xCT. In this study, NAC was used to reverify the effect of Mn in EAAC1 and xCT. ROS and GSH depletion induced by Mn was ameliorated by pretreatment with NAC whether *in vivo* or *in vitro*; moreover, cell activity and apoptosis caused by Mn were also significantly protected by NAC, which demonstrated that GSH depletion is the cause or result of cell death induced by Mn toxicity.

To further investigate oxidative damage induced by Mn and blocked EAAC1 and xCT transporters, several products of ROS reactions, such as MDA, sulfhydryl, carbonyl, and 8-OHdG, were measured. Zheng et al. suggest that MDA may serve as a useful biomarker for oxidative stress status after long-term, chronic exposure to welding fumes among career welders, as it can reflect lipid peroxidation and tissue injury resulting from oxidative damage [54]. We tested the MDA levels in the striatum to confirm lipid peroxidation induced by oxidative stress. Our results showed that the MDA levels in the striatum increased significantly after treatment with Mn. Mora et al. also found that high concentrations of Mn exposure in animals could produce free radicals and increase levels of MDA [55]. In our study, we found that Mn increased carbonyl and 8-OHdG but decreased sulfhydryl significantly. MDA, carbonyl, and 8-OHdG in the AAH + Mn or SSZ + Mn group were significantly higher than the group treated with 50 mg/kg Mn alone but lower in the NAC + Mn group. Meanwhile, sulfhydryl in the AAH + Mn or SSZ + Mn group rapidly reduced compared with the 50 mg/kg Mn group, while in the NAC + Mn group it increased greatly. The results suggest that Mn causes serious lipid peroxidation, protein modification, and DNA strand breaks in the striatum, in addition to inhibiting EAAC1- and/or xCT-enhanced oxidative stress induced by Mn.

In the brain, ultrastructural changes have been confirmed to be a common pathological reaction in neurodegenerative diseases, including Alzheimer's disease, PD, and manganism [56, 57]. The present study found that Mn exposure induced pathological injury of the striatum, exhibiting cell body swelling, interstitial vacuolation, abnormal tissue structure, and a decrease in the number of neurons. This result is consistent with the previous findings of Wang et al. [58]. In the AAH + Mn or SSZ + Mn group, the neuropathological injury was even worse; there was greater neuronal loss, more dead cells, and accompanying adipose degeneration. Electron microscopy showed there was a series of ultrastructure changes of neurons and astrocytes in the striatum after treatment with Mn, varying in the level of nucleolus collapse, membrane integrity damage, and intense chromatin condensation. Xu et al. have reported similar data in 2010 [59]. The ultrastructure alternation was worse in the AAH + Mn or SSZ + Mn group and included missing nucleus, unrecognizable organelle fragments, cytoplasm vacuolization formation, and balloon-like damage in the mitochondria. These findings showed that mitochondrial injury is the most common and serious injury of treatment with Mn or cotreatment with AAH or SSZ. These findings are consistent with reports that the highest Mn accumulation is in the astrocytic mitochondria [60]. Unfortunately, we have not clarified the relationship between mitochondrial injury and EAAC1/xCT inhibition in the striatum induced by Mn.

In summary, our data show that Mn exposure inhibited EAAC1 and xCT expression, induced oxidative stress *in vitro* and *in vivo*, and produced ultrastructure alternations in the striatum. Moreover, these findings suggest that dysfunction of EAAC1 and xCT may reduce GSH synthesis that can lead to a decrease in antioxidant ability. In conclusion, excessive exposure to Mn disrupts GSH synthesis through inhibition of EAAC1 and xCT to trigger oxidative damage in the striatum.

## Figures and Tables

**Table 1:** Primer sequences used for the amplification of each gene in this study.

Name	Oligo	Primer sequence
*β*-Actin	Sense primer	5′-CTACAATGAGCTGCGTGTGGC-3′
	Antisense primer	5′-CAGGTCCAGACGCAGGATGGC-3′
EAAC1	Sense primer	5′-ATGTGTCTGGGAAGATTGGTC-3′
	Antisense primer	5′-TTTACCCGTCCTGTTGATGTC-3′
xCT	Sense primer	5′-TTGCAAGCTCACAGCAATTCTG-3′
	Antisense primer	5′-CAGGTCCAGACGCAGGATGGC-3′

**Table 2:** Effects of Mn exposure and AAH pretreatment on ROS and GSH levels in neurons.

Group	ROS (*μ*mol/g pro)	GSH (*μ*mol/g pro)
Control	179.93 ± 3.61	26.85 ± 7.26
100 *μ*M MnCl_2_	191.79 ± 13.27	20.71 ± 4.11^∗^
200 *μ*M MnCl_2_	254.62 ± 6.85^∗∗^	17.72 ± 4.33^∗∗^
400 *μ*M MnCl_2_	324.48 ± 6.74^∗∗^	15.31 ± 3.08^∗∗^
250 *μ*M AAH + 400 *μ*M MnCl_2_	390.9 ± 12.38^∗∗^^##^	9.82 ± 1.64^∗∗^^#^
1 mM NAC + 400 *μ*M MnCl_2_	205.94 ± 6.09^∗∗^^##^	25.35 ± 5.88^#^

Data are mean ± SD; *n* = 6 each. ^∗^*p* < 0.05, ^∗∗^*p* < 0.01 denote statistical significance compared with the control group; ^#^*p* < 0.05, ^##^*p* < 0.01 denote statistical significance compared with the MnCl_2_ alone group.

**Table 3:** Effects of Mn exposure and SSZ pretreatment on ROS and GSH levels in astrocytes.

Group	ROS (*μ*mol/g pro)	GSH (*μ*mol/g pro)
Control	191.51 ± 3.36	23.49 ± 5.37
100 *μ*M MnCl_2_	199.62 ± 6.74	20.61 ± 6.72
200 *μ*M MnCl_2_	254.69 ± 9.74^∗∗^	17.97 ± 3.12^∗^
400 *μ*M MnCl_2_	296.46 ± 8.19^∗∗^	16.18 ± 3.11^∗∗^
200 *μ*M SSZ + 400 *μ*M MnCl_2_	324.86 ± 4.55^∗∗^^##^	10.16 ± 0.71^∗∗^^#^
1 mM NAC + 400 *μ*M MnCl_2_	218.49 ± 7.04^∗∗^^##^	20.61 ± 5.79^#^

Data are mean ± SD; *n* = 6 each. ^∗^*p* < 0.05, ^∗∗^*p* < 0.01 denote statistical significance compared with the control group; ^#^*p* < 0.05, ^##^*p* < 0.01 denote statistical significance compared with the MnCl_2_ alone group.

**Table 4:** Effects of Mn exposure and AAH or SSZ pretreatment on ROS and GSH levels in the striatum.

Group	ROS (*μ*mol/g pro)	GSH (*μ*mol/g pro)
Control	181.28 ± 7.21	31.25 ± 3.97
12.5 mg/kg MnCl_2_	335.34 ± 19.47^∗∗^	25.88 ± 6.15^∗∗^
25 mg/kg MnCl_2_	354.63 ± 8.71^∗∗^	19.07 ± 6.63^∗∗^
50 mg/kg MnCl_2_	390.18 ± 10.51^∗∗^	13.88 ± 3.39^∗∗^
500 mg/kg AAH + 50 mg/kg MnCl_2_	415.98 ± 12.44^∗∗^^#^	5.83 ± 1.19^∗∗^^#^
75 mg/kg SSZ + 50 mg/kg MnCl_2_	410.27 ± 9.38^∗∗^^#^	5.92 ± 1.5^∗∗^^#^
100 mg/kg NAC + 50 mg/kg MnCl_2_	344.73 ± 8.91^∗∗^^##^	19.84 ± 4.69^∗∗^^#^

Data are mean ± SD; *n* = 6 each. ^∗∗^*p* < 0.01 denote statistical significance compared with the control group; ^#^*p* < 0.05, ^##^*p* < 0.01 denote statistical significance compared with the MnCl_2_ alone group.

**Table 5:** Effects of Mn exposure and AAH or SSZ pretreatment on MDA, sulfhydryl, and carbonyl levels.

Group	MDA (nmol/mg pro)	Carbonyl (nmol/mg)	Sulfhydryl (*μ*mol/g)
Control	1.05 ± 0.42	3.08 ± 0.76	49.08 ± 6.18
12.5 mg/kg MnCl_2_	2.14 ± 0.57^∗^	5.8 ± 1.2^∗∗^	46.3 ± 7.45
25 mg/kg MnCl_2_	2.69 ± 0.76^∗∗^	7.48 ± 1.47^∗∗^	38.03 ± 2.12^∗∗^
50 mg/kg MnCl_2_	2.88 ± 0.88^∗∗^	8.19 ± 1.37^∗∗^	29.91 ± 7.08^∗∗^
500 mg/kg AAH + 50 mg/kg MnCl_2_	4.08 ± 0.99^∗∗^^#^	10.16 ± 1.72^∗∗^^#^	23.34 ± 3.09^∗∗^^#^
75 mg/kg SSZ + 50 mg/kg MnCl_2_	3.91 ± 0.71^∗∗^^#^	9.72 ± 0.94^∗∗^^#^	21.49 ± 4.92^∗∗^^#^
100 mg/kg NAC + 50 mg/kg MnCl_2_	2.03 ± 0.4^∗^^#^	6.19 ± 1.69^∗∗^^##^	39.02 ± 6.62^∗^^##^

Data are mean ± SD; *n* = 6 each. ^∗^*p* < 0.05, ^∗∗^*p* < 0.01 denote statistical significance compared with the control group; ^#^*p* < 0.05, ^##^*p* < 0.01 denote statistical significance compared with the MnCl_2_ alone group.

## Data Availability

The data used to support the findings of this study are available from the corresponding author upon request.
